# Compliance of public health facilities with essential medicines and health supplies redistribution guidelines in Mbale district, Eastern Uganda: a mixed-methods study

**DOI:** 10.1186/s40545-023-00545-0

**Published:** 2023-02-28

**Authors:** Immaculate Kyalisiima, Freddy Eric Kitutu, Linda Gibson, Immaculate Akaso, Amos Ndaabe, Herbert Bush Aguma, David Musoke, Richard Odoi Adome, Paul Kutyabami

**Affiliations:** 1 Department of Pharmacy, Makerere University School of Health Sciences, P.O Box 7062, Kampala, Uganda; 2 School of Social Sciences, Nottingham Trent University, Nottingham, UK; 3 School of Public Health, Makerere University, P. O. Box 7072, Kampala, Uganda

**Keywords:** Compliance, Essential medicines, Essential medicines and health supplies guidelines, Health facilities, Health commodities, Redistribution, Uganda

## Abstract

**Introduction:**

Redistribution of essential medicines and health supplies (EMHS) is a mechanism to address supply chain uncertainty by moving excess stock of health commodities from health facilities that are overstocked to health facilities with shortages, where it is most needed. It prevents the wastage of scarce resources and improves efficiency within a health supply chain system. Many public health facilities in Uganda experience stock-outs, overstocking, and expiry of essential medicines. This study assessed the compliance of public health facilities with the Uganda Ministry of Health redistribution strategy for EMHS in Mbale district, Eastern Uganda.

**Methods:**

A mixed-methods study was conducted among 55 respondents at public health facility level and five key informants at the district level. Audio-recorded data were transcribed and coded to develop themes. Thematic analysis was performed using ATLAS.ti Version 8.5. Quantitative data were analysed using IBM SPSS Version 24.0.

**Results:**

About a third (33%) of the surveyed health facilities complied with EMHS redistribution guidelines. Respondents agreed that EMHS redistribution had helped reduce health commodity expiries and stock-outs in health facilities. Respondents who did not know about the timely release of funds for redistribution were 68% less likely to comply, and those who said the guidelines were never shared were 88% less likely to comply with the guidelines.

**Conclusions:**

Compliance with the EMHS redistribution guidelines was low and associated with failure to share the guidelines with staff and inadequate awareness about release funds for EMHS redistribution. The district local government should allocate more funds to the EMHS redistribution.

## Background

Redistribution of Essential Medicines and Health Supplies (EMHS) is an essential mechanism in supply chains that allows public health facilities to move unused stock of health commodities that has not expired to where it is required [1]. This mechanism is essential for public health facilities in Uganda because of the inequity in EMHS allocations by the National Medical Stores (NMS) [2]. NMS is the government's central medical store mandated to procure, warehouse and distribute EMHS to all public health facilities. EMHS are procured using government funds from taxes and donors. Public health facilities are classified into seven levels based on the catchment population and services offered, namely: national referral hospitals, regional referral hospitals, referral hospitals, general hospitals, health centres (HCs) level IV, HCs level III, and HCs level II [3]. Higher level health facilities, HCs level IV and Hospitals order EMHS from the central medical store, NMS, every 2 months based on their allocated budgets. Lower level health facilities, HCs, level II and level III are allocated uniform kits per district [4]. Pharmaceutical kits contain selected EMHS in predetermined quantities for supply to lower level health facilities [5, 6].

Uganda is a low-income country with a GDP of $45.557 billion [7]. According to WHO, in 2020, the country spent 3.96% of its GDP on health [8]. Data on total pharmaceutical expenditure is not readily available [9]. The proportion of health expenditure allocated to medicines was estimated to be about 30%. Health expenditure is majorly from the government, with donors providing the largest share of funding. In addition, out-of-pocket payments and private insurance are significant sources of health care funding [10]. The annual budget for medicines in the public sector in FY 2020/2021 was UGX446.4 billion, representing about 15% of the health sector budget. The health budget as a proportion of the national budget was only 6.1%, falling short of the 15% recommended by the Abuja Declaration [11]. The annual budget for EMHS is far below the estimated national need for medicines in public health facilities, where most patients seek medical care [12]. Moreover, facilities at the same level are allocated the same budgets, yet their patient loads differ widely [2, 13]. This results in varied availability of EMHS characterized by excess stock and stock-outs in health facilities [10].

Redistribution of EHMS promotes optimal use of available health commodities by providing a platform to move them from health facilities they are less needed or overstocked to health facilities in greater need with under-stocking. As such, it prevents EMHS shortages and mitigates wastage in health facilities. These EMHS are an integral part of health service delivery and should always be available if the Ministry of Health’s (MOH) targets for service delivery are to be met. However, many public health facilities in Uganda continue to report stockouts, expiries, and only about 40.6% achievement of set targets in managing endemic diseases, such as malaria [4, 11, 14, 15]. The recent annual health sector performance report showed that only 43% of the health facilities had 95% availability of a basket of commodities over a 3-month period which is below the target of 75% of the facilities [11]. It is also not uncommon to find health facilities overstocked with some medicines [2]. All this indicates sub-optimal health supply chain system performance and that redistribution of EMHS is not being done appropriately in these facilities to mitigate frequent stock-outs [9]. Maldistribution is attributed to weak supply chains resulting from defective inventory practices, such as poor stock monitoring, deficiency of knowledge of basic expiry prevention tools, low participation of clinicians, profit- and incentive-based quantification, and irrational use of medicines [16, 17]. The low availability of EMHS undermines the efforts to meet the targets of the MOH and the World Health Organization for equity and efficiency [16, 18]. Reduced availability results in reduced access to health care, increased out-of-pocket expenditure as patients must look for alternatives, medical errors, non-adherence to treatment, drug resistance, and increased morbidity and mortality [17, 19]. Supply chains of EMHS, therefore, need to be strengthened as they come with a lot of uncertainty and interruptions impacting performance and efficiency of the health system. Mechanisms, such as redistribution, when effectively implemented, optimise the use of available resources and improve the efficiency with which health systems work. In countries, such as the United States of America, redistribution of medicines has been utilized to reduce medicines wastage and leads to great cost avoidance [20].

Redistribution is important in bridging gaps in service delivery. However, redistribution can be challenging as it requires proper stock management and collaborative action among decentralised actors of the health supply chain to ensure efficient delivery of EMHS [21]. To streamline and harmonise redistribution, the MOH developed the National Redistribution Strategy for the Prevention of Expiries and Handling of Expired Medicines and Health Supplies (EMHS) in 2012 and revised it in 2018 [1, 22]. The strategy was to avoid EMHS wastage through reported paradoxes of excess stock in one facility, while another has a stock out within the same district. The stock of a specific medicine is considered to be excess when the months of stock are more than four on any specific day. However, on numerous occasions, health workers have been noted as reluctant to follow the guidelines to redistribute [1]. An unpublished report of a survey done in Uganda found compliance with the 2012 redistribution guidelines to be 39% [23]. The survey, however, did not include any of the districts in the Bugisu sub-region. Mbale district is the regional capital of Bugisu and has experienced a decline in health services performance on the district league table of MOH. Therefore, this study assessed the compliance of health facilities within Mbale district with the redistribution guidelines and explored factors associated with non-compliance to the redistribution guidelines. The study findings are instrumental in identifying gaps contributing to compliance with the EMHS redistribution guidelines, which is critical for decision-making.

## Methods

### Study design and setting

We used a mixed-methods cross-sectional study design. Quantitative data was used to determine compliance and identify-associated factors. Qualitative data were collected simultaneously to supplement the factors associated with compliance. The data collected were from primary care facilities and administrative staff at district level in Mbale from June to August 2020.

Mbale district is one of the districts found in the mid-eastern region of Eastern Uganda. It is divided into three constituencies: two counties (Bungokho south and north) and Mbale municipality, with its largest city being Mbale city. Mbale district has 36 public health facilities, of which 35 are primary care facilities, and one is a regional referral hospital that serves the eastern region [3]. The district had an estimated population projection of 586,300 people in 2020 [24]. The district was chosen for the study because of its huge decline in health sector performance from being in the top 10 districts in 2016/2017 to 34th and 45th in 2017/2018 and 2019/2020, respectively [25–27].

### Study population

Our study units comprised primary care health facilities in Mbale district from which respondents were purposively selected. We included in the study all primary care facilities funded by the government. The selected facilities were from health centre levels II, III, and IV. HCs levels I, II, III, and IV are the only facilities in the healthcare system that provide primary care in Uganda [2, 28]. They deliver the first connection between the public and the formal health sector and focus mainly on infectious disease prevention and treatment services. Village Health Teams (VHTs) constitute health centre I and are attached to a nearby health facility [29].

HCs level IV are mandated to serve a target population of 100,000 people and have provisions for an operating theatre, inpatient, and laboratory services, and act as a referral facility for HCs level III in their jurisdiction. HCs level III have a target population of 20,000 people and have provisions for basic laboratory services, maternity care, and inpatient care. HCs level II, on the other hand, are lower level facilities and are mandated to serve a target population of about 5,000 people providing outpatient services and outreach programs only [28].

The health facilities are staffed differently based on their level of care, determined by the MOH. HCs level IV have between 10 to 21 staff, which include; 1 doctor, clinical officers, qualified nurses, midwives, and nursing aides. HCs level III have 10 to 6 staff, including clinical officers, qualified nurses, midwives, and nurse aides. HCs level II, as lower level facilities, have approximately four staff including a qualified nurse, midwife, and nurse aides [28]. Specific cadres are employed depending on the services to be offered across the different facility levels, although currently, staffing levels stand at only 45%, 20%, and 13% of HCs levels II, III, and IV, respectively [11]. In addition to the staffing constraints, nationally, HCs levels IV, III and II received 2.6%, 6.3%, and 2.3%, respectively, of the National Medical Store (NMS) expenditure in the 2020/2021 financial year [11].

Ordering of medicines follows a pull system at Hospitals and HCs level IV and a push or kit system at HCs level III and II [4]. The pull system requires each health facility to determine what and how much to order. On the other hand, the push system requires each health facility to receive predefined kits [5]. The health facility in charge approves bi-monthly orders for medicines at each facility before submission. A graduate pharmacist makes orders at the hospital level, dispensers with qualifications of diploma in pharmacy at HCs level IV, and stores in charge at HCs level III and II. Annual facility budgets are divided into six cycles to approximate the value of commodities ordered every cycle. The quantity to order each month is calculated using a formula incorporating the quantity on hand and maximum stock. When the total amount exceeds the cycle budget, vetting commodities is done using VEN classification to prioritize the vital items [30].

### Study sample size

We required a sample size of at least 30 health facilities according to the WHO guidelines on survey of health facilities [31]. The sample size for quantitative data was determined using Yamane’s proportionate method, since the district had a finite population size. This sampling method was used to combine responses into categories and sample size based on proportions [32].

The proportions of each of these facilities were used to determine the exact number of participating facilities. Once the number had been determined, these were then randomly sampled. The sample size was calculated using the formula below based on a finite number of facilities.

$$n = \frac{N}{{1 + N\left( e \right)^{2} }}$$

$$n = {{\left( {35} \right)} \mathord{\left/ {\vphantom {{\left( {35} \right)} {1 + 35\left( {0.05} \right)^{2} }}} \right. \kern-0pt} {1 + 35\left( {0.05} \right)^{2} }} = 33$$

where n is the sample size, *N* is the population size, and *e* is the margin of error.

Proportions for each level of facility in the study area were (3 HCs level IV (8.6%), 23 HCs level III (65.7%), and 9 HCs level II (25.7%)). Therefore, 3 HCs level IV, 22 HCs level III, and 8 HCs level II were sampled. The sample size of respondents at health facility level was calculated based on the assumption that a facility had one in charge and one store in charge. The total number of respondents was, therefore, 66. The District Health Officer (DHO) and four Medicines Management Supervisors (MMS) were purposively selected for key informant interviews. MMSs are health workers that provide supportive supervision to improve medicines management in public health facilities [33, 34].

### Variables

The outcome variable of the study was compliance with EMHS redistribution guidelines. This was a binary outcome, where a facility would be compliant or non-compliant. A facility was reported compliant if it scored equal to or above an arbitrary cutoff of 80% on the observational checklist. The independent variables were categorical and included constructs of the EMHS redistribution guidelines, including the steps and triggers of redistribution. Others were the factors that affect stock levels and redistribution at health facilities, the availability of funds, bureaucratic processes, knowledge of staff, up-to-date stock cards, availability of EMHS, and communication channels.

### Data collection

Three data collection tools were used in the study, including a questionnaire, a key informant guide, and an observational checklist. The tools were distributed among the respondents that had been purposively selected.

The tools were administered by the principal investigator together with two research assistants. The assistants had a minimum qualification of a bachelor’s degree and were trained on data collection procedures before commencement of the exercise. A pilot study was conducted in 5% of the calculated sample size before data collection to pre-test the data collection tools.

Administrative clearance was obtained from the offices of the Chief Administrative Officer (CAO), the DHO, the town clerk, and the manager responsible for each health facility. On arrival at the facility or district office for the study, the researchers introduced themselves and stated their reason for the visit. The researchers found the selected respondents at their respective workstations between 8:30 am and 4:30 pm. They were given introductory letters, briefed about the study being conducted, given consent forms, and allowed to participate freely without coercion. Only respondents that gave informed consent were allowed to participate in the study. The interviews ran for about 30–45 min each, and these were supplemented with on-site observations using an observational checklist.

### Quantitative data collection

Quantitative data was gathered using a questionnaire and checklist. The questionnaire was used to collect data from respondents at the health centres to determine their socio-demographic characteristics and possible factors associated with non-compliance. The questionnaire was administered by research assistants and had both open and closed-ended questions allowing the respondents to explain where necessary. The questions were formulated based on the activities involved in the process of redistribution as stated in the guidelines, and some questions were adapted from a similar questionnaire used to carry out a scoping study about compliance with redistribution guidelines [1, 23]. Questions asked included knowledge of the steps of redistribution and its financing, if the facility had excess stock, if the guidelines were available and if they had ever been trained. Probing for interesting responses and observation of nonverbal responses were also done.

The observational checklist supplemented the responses and was guided by triggers, including; A facility has an excess of one EMHS, while another has a deficit, the facility's stock is expected to expire before being used, the facility has EMHS distributed to it in error, especially when a lower level facility received supplies meant for a higher level facility and facility has more EMHS of a short shelf life than what was forecasted for use.

While using the checklist, we reviewed copies of the issue and requisition vouchers and stock records. We checked for occurrences of excess stock or deficits, availability of the guidelines, and the use of any communication system to alert departments. A percentage score was computed from the scores among the facilities that experienced triggers of redistribution to determine compliance.

### Selection of tracer commodities

We used documents of six tracer items to investigate compliance, including Artemether/Lumefantrine tablets, isoniazid tablets, cotrimoxazole tablets, oxytocin injections, metformin tablets, and rapid diagnostic test kits for malaria. We selected two tracer commodities, Artemether/Lumefantrine and malaria rapid diagnostic test kits because of the high burden of malaria and the large number of people at risk of getting the disease [35, 36]. Isoniazid and cotrimoxazole were selected for their use among HIV patients for preventive treatment against tuberculosis and as prophylaxis against opportunistic infections. We also selected metformin because of the increasing burden of Diabetes Mellitus and oxytocin because of its importance in managing obstetric conditions [35, 37].

### Qualitative data collection

We used a key informant interview guide to conduct interviews with the DHO and the MMSs, who had the most information about the overall redistribution process in the district. These interviews increased our understanding of the findings as they had extended probing. Open-ended questions were used during the interviews, and the sessions were recorded using a mini digital voice recorder to reduce the likelihood of omitting relevant information.

### Data quality control and management

To ensure the quality of data collected was assured, the tools used were pre-tested on two health facilities in a similar setting before their use in the field. The tools were then revised based on the feedback to ensure that these would be comprehended and appropriate responses obtained from the respondents.

The research assistants were also trained before data collection to ensure that all relevant information was captured. They were trained on the study objectives, how to use the audio recorders and the ethics of working when interacting with the respondents. Completeness of data was ensured by checking filled tools in real time to identify any missing data.

### Data analysis

Quantitative data was transferred from a Microsoft Excel 2016 spreadsheet (Microsoft Corporation, Washington, USA) and entered into Epi-Info version 3.5.1(CDC, Atlanta, Georgia). to be checked for consistency. Cleaned data was then exported to IBM SPSS Version 24.0 for analysis. Descriptive statistics were determined for the population and respondents from the univariate analysis. Test for association was done using logistics regression through multivariate analysis. Associations with the diverse factors were determined using odds ratios, p values, and a confidence interval of 95%.

Qualitative data were analysed using ATLAS.ti version 24.0(Scientific Software Development GmbH, Berlin, Germany) Thematic analysis was utilized. Qualitative data collected from recordings of interviews were transcribed, and a codebook was created for the different variables. The coded qualitative data were then categorized and grouped into themes for analysis. Continuous theme searching and reviewing were done until no new codes were observed from the scripts.

## Results

### Background characteristics

We visited a total of 33 public health facilities in Mbale district and had a response rate of 98%. We were only able to meet 55 respondents instead of the intended 66 respondents at the health facilities, because some health facility in-charges also served the role of the store in-charges. In addition, one store in-charge was on maternity leave and, therefore, could not be interviewed (Table 1).

**Table 1:** Background information of the respondents at the selected health facilities

Characteristics	Frequency (*n*)	Percentage (%)
Level of the health facility		
Health centre IV	5	9.1
Health centre III	38	69.1
Health centre II	12	21.8
Professional qualifications of the respondent		
Nursing cadre	19	34.5
Clinical Officer	25	45.5
Medical officer	8	14.5
Other	3	5.5
Sex of the respondent		
Male	24	43.6
Female	31	56.4
Years of services		
5–10	10	18.2
10–15	27	49.1
> 15	18	32.7
Position of the respondent at the health facility		
Facility in-charge	41	74.5
Stores in-charge	11	25.5

The majority (69.1%, *n* = 38) of respondents were from health centre III, and about half of the respondents (45.5%, *n* = 25) were clinical officers by cadre. More than half (56.4%, *n* = 31) of the respondents were female, and nearly half (49.1%, *n* = 27) had been in services for 10–15 years. Most (74.5%, *n* = 41) of the respondents were health facility in-charges.

In addition, we interviewed five key informants, four of whom were male, three had worked for more than 15 years, and three were clinical officers by cadre.

### Compliance with the EMHS redistribution guidelines

The majority (67.3%, *n* = 37) of respondents noted that the health facility did not have guidelines on redistribution available, and only (30.9%, *n* = 17) said the guidelines being used were up-to-date. Only (29.1%, *n* = 16) respondents agreed that guidelines were always used, and the information was verified with copies of vouchers and stock cards filled during redistribution at the facility. In addition, these respondents also confirmed that guidelines were always used in case there was a trigger (excess stock) to redistribute. The respondents’ information was verified by reviewing signed copies of the health facility's HMIS 017 vouchers and stock records.

Compliance of the health facilities was determined from the percentage scores of each facility that had excess stock. About one-third of health facilities (33.3%) were found compliant.

### Triggers of redistribution

Key informants agreed with the respondents at the facility that redistribution took place only when necessary. The respondents also noted that communicating excess stock at the health facility was done to the DHO through the health facility in charge.

*“In case of excess, I call the DHO, they have medicines management supervisors who inform us on what to do, or we take for redistribution. There are forms that we fill in all drugs in excess we take them to the district, and they also send messages using mTrac” HF-05*

However, the key informants stated that sometimes the drugs were brought to the district stores when they were about to expire and would not serve the intended purpose.

*“Sometimes the drugs come when it's late; they calculate and find that some drugs may not be consumed.” KII-05*

### Following the redistribution steps in the guidelines

When asked about the steps of redistribution, the responses were mixed as more than half 30 (54.5%) did not know about the existence and the content of the guidelines., However, they knew there was a procedure to follow in cases of excess and deficits to avoid expiry.

*“The guidelines are not properly disseminated because, at first, they were between facilities to facilities. It was not properly emphasized, and not everyone perceives it seriously if not followed by mTrac.” HF-10*

Even though most 39 (70.9%) respondents thought that knowledge of the guidelines was not well-disseminated, all key informants did not seem to agree.

*"Every facility has a copy of the guidelines, but you know when you ask the staff, and you ask, they claim they don't have, but when you check, you find the books stacked in a corner full of dust” KII-01*

All the key informants agreed that the redistribution procedures were clear and that facilities were making an effort. The process was long and required individuals to use their funds.

*“….the process is generally tedious and might even cost patients their lives.” KII-02*

*“Those steps are manageable……due to the length of the procedure, it makes the guidelines a bit complicated for some people, and at the end of the day, you find in charges saying To Whom It May Concern the drugs are over so let's wait for the next consignment.” KII-01*

### Factors associated with compliance with redistribution guidelines

Respondents who did not know if the money required for redistribution was released on time were 67.6% (*p* = 0.007, 95% CI 0.127–0.685) less likely to comply with the redistribution guidelines than those who said the money required for redistribution was never released on time. Respondents who said the guidelines are sometimes shared with the staff in other departments were 92% (*p* = 0.037, 95% CI 0.097–0.0264) less likely to comply with the redistribution guidelines than those who said the guidelines are always shared with the staff in other departments. Similarly, respondents who reported that the guidelines were never shared with staff in other departments were 88% (*p* = 0.003 95% CI 0.097–0.0264) less likely to comply with the redistribution guidelines than those who said the guidelines were always shared with the staff in other departments (Table 2).

**Table 2:** Factors associated with compliance with the EMHS redistribution guidelines

Variables	OR (95% CI)	*p* value
Money is required to re-distribute released on time		
Never	1.0	
I don’t know	0.324 (0.127–0.685)	0.007
Guidelines were shared with staff in other departments		
Always	1.0	
Sometimes	0.080 (0.061–0.109)	0.037
Never	0.120 (0.097–0.0264)	0.003

### Other factors affecting compliance with redistribution guidelines

#### Transportation costs and delivery

The biggest challenge faced during redistribution was the cost of transporting the medicines and supplies, leading to delays in identification and movement. Funds for redistribution were not readily available.

*“One of the key constraints is money. Most facilities don't have money when needed. The PHC and RBF money comes, but many activities need the money, and, in most cases, redistribution is not one of those activities*.” *KII-01*

*“In cases where a facility is far away from the district, staff start grumbling asking why they should put in their money.” KII-01*

*“Financially, there's a big challenge if the partners withdrew, we might have interruptions” KII-05*

However, some respondents believed that if people responsible for resources at the facility used them appropriately, they would not have transportation challenges.

“*Some health facilities do not appreciate what they have. If I run out of stock and I need to get drugs from another facility when all the vouchers are signed, I hire a means of transport to get the drug to me because the facilities always have money” HF-10*

#### Bureaucracy involved in the process of redistribution

More than half, 29 (52.7%) of the respondents, thought that the required signatures on the notification forms were too many, with some not being stationed at the facilities making the process long. In addition, respondents agreed that the process was too long.

*“People who sign are many…., sometimes the DHO is very busy to sign, someone can keep on coming until they give up” HF-02*

#### Inconsistency in the delivery process

Inconsistencies in the delivery of drugs and supplies and failure to inspect items contributed to non-compliance with the EMHS redistribution guidelines. Respondents stated that the NMS and Joint Medical Stores (JMS) continuously delivered what was not requested by the facilities.

*“The uncertainty of deliveries where someone thinks that if I give out this and this, they are not very sure that the next cycle they will receive that item from NMS so at times they end up not redistributing” KII-03*

#### Knowledge gaps among the healthcare workers

The respondents believe that the health workers were not well conversant with the stock management and the redistribution guidelines as many had not been trained on these, hindering the process.

*“Now, some staff do not know what to do with physical counts and do not check for expiry date. Sometimes physical counts are done and even include drugs that are expired” KII-02*

The key informants also noted that on top of most facilities not having personnel trained to manage stores, the few who train are constantly transferred to carry out other roles.

*“…after we have mentored somebody for a month, you come back and find when a person has been transferred. There are frequent transfers, so you retrain.” KII-03*

*"You train today, get an action point, and the person is transferred." KII-02*

The key informants also noted that they trained, but the staff did not improve.

*“We have taken the health workers through the given guidelines, but they don’t want to understand” KII-04*

#### Attitude of the health workers and facility person’s in-charge

Most of the key informants and some respondents noted that the attitude of the staff towards stock ownership also affected redistribution as some regarded it as personal property, causing them to hold onto the excess items. Others noted that some health facilities did not take the responsibility to carry out proper stock-taking, making it hard to plan.

*"People forget that these are government drugs and then think that the drugs are the facility's drugs, so there is that rigidity in releasing the excess." KII-03*

*"When you give some in-charges the guidelines, they just keep them in their bags, which creates a problem." KII-04*

#### Individual responsibility

The respondents also noted that some in-charges did not take the initiative to carry out necessary activities to prevent the accumulation of excess stock and expiries. They cited being overloaded with work as a hindrance to paying close attention to taking appropriate action on redistributable stock in the facility store.

*"Then appointed people may get overwhelmed with work and not get time to go to the store, and sometimes drugs might expire without their knowledge." KII-03*

## Discussion

Our findings showed that compliance with the EMHS redistribution guidelines was at only 33.3% and was associated with failure to disseminate guidelines and the lack of knowledge on the timely release of money for redistribution. This study is important, because no published literature on compliance with redistribution guidelines exists. The study findings are of major importance to districts in devising strategies to address stock-outs, overstocking and expiries of essential medicines in public health facilities.

Only a third (33.3%) of the health facilities complied with the EMHS redistribution guidelines. These findings are comparable to those from a nationwide scoping review done in Uganda by Sematiko, where compliance with the guidelines was as low as 39% [23]. However, the findings differ from another study that found that 75.9% of public health facilities in Uganda used redistribution to cope with stock-outs [4]. The difference in findings could be due to a social desirability bias leading to an overestimation of redistribution. Failure to comply with the guidelines undermines efforts by the government to fight expiries, stock-outs, and their undesirable consequences. Indeed, a study by Zakumumpa et al. showed redistribution as a strategy to fight chronic stock-outs among health facilities [14]. The low compliance in the present study implies that even when redistribution triggers are present, such as excess stock, while another has a deficit, facility in-charges and staff still hold back from following the redistribution guidelines.

In this study, we found that respondents who reported that the guidelines were never shared with staff in other departments were 88% less likely to comply. Failure to share guidelines limits the knowledge circulation about redistribution among staff. This finding corresponds with a study by Srivastava and Singh to identify the antecedents and consequences of integrated supply chain performance in healthcare systems. The study showed that knowledge sharing among staff impacted the performance of the supply chain [38]. Another study done in Iran on the role of information sharing on supply chains showed that information sharing positively affected the integration, efficiency and performance of drug supply chains [39].

Failure to share guidelines further led to staff knowledge gaps, as over half (54.5%) of the staff did not know about the guidelines, even though key informants stated that they gave out guidelines and trained staff. The knowledge gap affects stock management practices and compliance with the guidelines. This finding is supported by a study done in Malawi to investigate the management of drug supplies for life-threatening diseases, which showed that training in the management of medicines affected their availability [40]. Gaps in knowledge affect the ability of staff to appropriately quantify and make projections when planning, further causing mal-distribution and expiries, as pointed out in a study done in Uganda [41]. In our study, the knowledge gap was further attributed to the constant transfer of staff and the recruitment of staff that are not qualified to carry out the relevant assigned roles in the management of EMHS. We found that this led to staff including expired medicines in the physical counts.

In addition, we also found that compliance was associated with staff not knowing if money was released on time. The failure to share information on availability of funds among health facility staff means that few staff knew about the party meant to incur the cost of redistribution. This implies that staff will become more reluctant to redistribute as no money at the facilities is allocated, and they will rely on implementing partners. Previous studies showed that redistribution was expensive and required additional funding to be effected [2, 6]. In the guidelines, no budgetary allocations are made to cater for transportation. A study conducted in Elgeyo Marakwet County, Kenya, to assess factors affecting supply chain efficiency in public health facilities showed that healthcare workers agreed that transport coordination affected their supply distribution [42]. Timely access to transport facilitation for redistribution of medicines is, therefore, essential.

We also found that redistribution was bureaucratic to avoid the occurrences of theft and to streamline the process. The lengthy procedures affected redistribution as the respondents felt that the approval process was tedious, discouraging many from initiating it. This finding also agrees with the study by Sematiko, where all the respondents agreed that the long bureaucratic process affected redistribution [23]. A study by Mikkelsen-Lopez et al. to determine the pattern of availability of Artemisinin Combination Therapy (ACT) and possible causes of stock-outs in public health facilities in Tanzania found that the long bureaucratic processes were a major factor in reducing availability of stock [43]. There is a need to minimize the approval process to ease redistribution of excess stock.

We also found that inconsistencies in the delivery of EMHS at health facilities also affected compliance with the guidelines and increased practises like hoarding EMHS stock that may be required at another health facility due to the uncertainty created. Some health facilities intentionally did not declare excess stock, because they were unsure if they would get supply in the next cycle from NMS. In a study to understand the problems underlying drug shortages, distribution uncertainties were seen to contribute to more shortages as some facilities stocked items that were disproportionate to their demand [44]. Our findings align with a study done in Malawi to investigate the management of drug supplies for life-threatening diseases. In the study, deficient deliveries from regional medical stores and uneven distribution of drugs among health centres contributed to inadequate EMHS supplies [40]. The NMS should stick to delivery schedules of medicines to reduce uncertainty among health facilities.

The study sought to learn from the health workers involved in redistribution of medicines. The study's strength is that it employed mixed methods, which enabled us to understand compliance with redistribution guidelines in-depth. The study also had limitations. First, some of the health workers approached had concerns about being reprimanded because of the information provided. Reassurance of the respondents’ confidentiality was done to mitigate this challenge. Second, during data collection, some health facilities had improperly filled stock cards and the requisition and issue vouchers which affected some of the data to support determination of the level of compliance. Third, some of the assessment of the items for measuring compliance was based on self-reports by the respondents, hence a possibility of social desirability bias.

## Conclusions

Compliance with the EMHS redistribution guidelines was low. Several hindrances to compliance with redistribution guidelines were cited. Key hindrances were lack of guidelines, failure to share the guidelines with staff and not knowing about releasing money for redistribution. To improve compliance with redistribution guidelines, funds for transporting medicines should be budgeted for at the district. Health facility in-charges should sensitize staff working in stores about the redistribution process. The DHO should prioritize regular supervision of health facilities with an emphasis on preventing medicine stock-outs and overstocking.

## Data Availability

The data sets generated and analysed during this study are available from the corresponding author upon reasonable request.

## Abbreviations

ACT: Artemisinin Combination Therapy
DHO: District Health Officer
EMHS: Essential Medicines and Health Supplies
HC: Health Centre
JMS: Joint Medical Stores
MOH: Ministry of Health
MMS: Medicine Management Supervisor
MSH: Management Sciences for Health
NMS: National Medical Stores
SHS-IRB: School of Health Sciences Institutional Review Board
SPSS: Statistical Package for Social Scientists
